# Cardiometabolic Effects of Denosumab in Premenopausal Women With Breast Cancer Receiving Estradiol Suppression: RCT

**DOI:** 10.1210/clinem/dgae003

**Published:** 2024-01-05

**Authors:** Sabashini K Ramchand, Rudolf Hoermann, Shane White, Belinda Yeo, Prudence A Francis, Cecilia L H Xu, Jeffrey D Zajac, Ego Seeman, Mathis Grossmann

**Affiliations:** Department of Medicine, Austin Health, University of Melbourne, Victoria 3084, Australia; Department of Medicine, Massachusetts General Hospital, Harvard Medical School, MA 02114, USA; Department of Medicine, Austin Health, University of Melbourne, Victoria 3084, Australia; Department of Medicine, Austin Health, University of Melbourne, Victoria 3084, Australia; Olivia Newton-John Cancer & Wellness Centre, Austin Health, Victoria 3084, Australia; Department of Medicine, Austin Health, University of Melbourne, Victoria 3084, Australia; Olivia Newton-John Cancer & Wellness Centre, Austin Health, Victoria 3084, Australia; Peter MacCallum Cancer Centre, Sir Peter MacCallum Department of Oncology, University of Melbourne, Victoria 3052, Australia; Department of Medicine, Austin Health, University of Melbourne, Victoria 3084, Australia; Department of Medicine, Austin Health, University of Melbourne, Victoria 3084, Australia; Department of Endocrinology, Austin Health, Victoria 3084, Australia; Department of Medicine, Austin Health, University of Melbourne, Victoria 3084, Australia; Department of Endocrinology, Austin Health, Victoria 3084, Australia; Department of Medicine, Austin Health, University of Melbourne, Victoria 3084, Australia; Department of Endocrinology, Austin Health, Victoria 3084, Australia

**Keywords:** denosumab, body composition, glucose metabolism, lipids, breast cancer, estradiol suppression

## Abstract

**Context:**

Menopause is associated with changes in musculoskeletal, body composition, and metabolic parameters that may be amplified in premenopausal women receiving estradiol suppression for breast cancer. Denosumab offsets deleterious skeletal effects of estradiol suppression and has been reported to have effects on body composition and metabolic parameters in preclinical and observational studies, but evidence from double-blind randomized controlled trials is limited.

**Objective:**

To assess the effect of denosumab on body composition and metabolic parameters.

**Methods:**

In a prespecified secondary analysis of a 12-month randomized, double-blind, placebo-controlled trial, 68 premenopausal women with breast cancer initiating ovarian function suppression and aromatase inhibition were randomized to denosumab 60-mg or placebo administered at baseline and 6 months. Outcome measures were total and regional fat and lean mass (DXA), body mass index (BMI), waist and hip circumference, fasting glucose, HOMA-IR, and lipid profile. Using a mixed model, between-group mean adjusted differences over time are reported.

**Results:**

Over 12 months, relative to placebo, android and gynoid fat mass decreased in the denosumab group (−266 g [95% CI −453 to −79], *P* = .02, and −452 g [−783 to −122], *P* = .03, respectively). Total fat mass and waist circumference were lower in the denosumab group but not significantly (−1792 g [−3346 to −240], *P* = .08 and (− 3.77 cm [−6.76 to −0.79], *P* = .06, respectively). No significant treatment effects were detected in lean mass, BMI, hip circumference, fasting glucose, HOMA-IR, or lipid profile.

**Conclusion:**

In premenopausal women receiving estradiol suppression, denosumab decreases some measures of fat mass with no detectable effects on other measures of body composition or metabolic parameters.

In premenopausal women with early-stage estrogen receptor (ER)-positive breast cancer at high risk of disease recurrence, maximal suppression of circulating estradiol using ovarian function suppression and aromatase inhibition improves oncological outcomes compared to standard therapy with tamoxifen, a selective estrogen receptor modulator (1, 2).

The menopausal transition is associated with an increase in abdominal fat and dysregulation in glucose and lipid homeostasis (3-6), adverse features that increase the risk of cardiovascular disease. Combined ovarian function suppression and aromatase inhibition precipitously decreases circulating estradiol to near zero, concentrations below that seen in women transitioning through natural menopause (7). For this reason, the unfavorable changes in body composition and metabolic parameters observed in postmenopausal women may be exaggerated when early menopause, an independent predictor of cardiovascular disease, is induced in premenopausal women treated with maximal estradiol suppression therapy (8, 9). Given the improved survival benefit of estradiol suppression therapy in premenopausal women with early-stage breast cancer but increased cardiovascular morbidity and mortality (10, 11) in survivors of breast cancer, attention to intermediate and longer-term surrogate endpoints for cardiovascular risk is needed in order to maximize the benefit of an effective treatment for breast cancer.

Denosumab, a fully humanized monoclonal antibody that inhibits the receptor activator of nuclear factor kappa-B ligand (RANKL) produces almost complete and sustained suppression of bone remodeling (12). This treatment is used in women with breast cancer receiving aromatase inhibitors to reduce the risk of fragility fractures (13, 14). While the focus of denosumab therapy has been on bone health, there is evidence to suggest a possible beneficial effect of RANKL inhibition on body composition and metabolic parameters, in particular, glucose homeostasis. For example, in animal models and in observational studies of postmenopausal women with type 2 diabetes, RANKL inhibition is associated with improved glucose metabolism (15-21). In a recent population-based cohort study of primary care patients from the United Kingdom, a decreased incidence of type 2 diabetes was observed in adults with osteoporosis treated with denosumab compared to those treated with oral bisphosphonates (22). While several other studies support a beneficial effect of RANKL inhibition on glucose and other metabolic parameters (23, 24), other studies do not (25-29). However, evidence from prospective randomized controlled trials (RCTs) examining the relationship between denosumab and body composition and metabolic parameters is limited.

We therefore examined prespecified secondary outcomes, including fat mass, lean mass, body mass index (BMI), waist and hip circumference, glucose homeostasis, and lipid profile, in a randomized double-blind placebo-controlled trial of premenopausal women undergoing maximal estradiol suppression, the primary aim of which was to evaluate the efficacy of denosumab in preventing bone loss.

## Methods

### Study Design and Participants

A detailed description of the study design and participant characteristics have been reported in the primary paper (submitted). In brief, we conducted a 12-month randomized, double-blind, placebo-controlled trial at a single academic hospital in Australia, to assess the efficacy of denosumab in preventing bone loss and microstructural deterioration in premenopausal women with early-stage breast cancer initiating ovarian function suppression and aromatase inhibition. Participants were stratified by lumbar spine bone mineral density (BMD, < or > 1.09 g/cm^2^) and BMI (< or > 27 kg/m^2^) and then randomized with equal probability to receive denosumab 60-mg or placebo subcutaneously every 6 months.

Premenopausal women aged 18 to 55 years were eligible for inclusion if they had histologically confirmed early-stage ER-positive breast cancer and were intended for combined ovarian function suppression (use of gonadotropin releasing hormone [GnRH] analogs or bilateral oophorectomy) and aromatase inhibition. Women were defined as being premenopausal if they had a regular menstrual cycle in the 3 months prior to their diagnosis of breast cancer.

The main exclusion criteria were ovarian function suppression for longer than 12 weeks at the time of trial inclusion, any previous use of a selective estrogen receptor modulator or bone targeted therapy, glucocorticoid use for more than 14 days within 6 months of enrollment, a BMD T-score < −2.0 SD at the spine or hip, metabolic bone disease, or a history of minimal trauma fractures. Participants were also excluded if they had 25-hydroxy vitamin D concentration <12 nmol/L, diabetes mellitus, malignant disease (apart from breast cancer and nonmelanoma skin cancer), history of any solid organ or bone marrow transplant, major systemic disease, pregnancy or breastfeeding, excessive alcohol intake, major psychiatric history, osteonecrosis of the jaw, or atypical femoral fractures.

The study protocol was approved by the Austin Health Research Ethics Committee and preregistered with the Australian New Zealand Clinical Trials Registry (anzctr.org.au), identifier ACTRN12616001051437. All study participants provided written informed consent before inclusion in the study.

### Study Outcomes

The primary endpoint of the study and the basis for *a priori* power estimates was total volumetric BMD at the distal tibia. Distal tibia and distal radius volumetric BMD and bone microstructure outcomes measured using high-resolution peripheral quantitative computed tomography (HR-pQCT), and spine and hip areal BMD outcomes measured using dual-energy x-ray absorptiometry (DXA), have been reported elsewhere (submitted). Here we report the prespecified secondary endpoints of total and regional fat mass and lean mass measured by dual-energy x-ray absorptiometry (DXA), anthropometric measures (body weight, BMI, waist and hip circumference, and waist/hip ratio), measures of glucose metabolism (fasting plasma glucose, glycated hemoglobin [HbA1c], fasting insulin and C-peptide concentrations, and homeostatic model assessment of insulin resistance [HOMA-IR] derived from measurements of fasting plasma glucose and serum insulin (30)), and lipid profile.

### Assessments

Study visits were conducted at 0, 3, 6, and 12 months. Fasting morning blood samples for glucose, HbA1c, insulin, and C-peptide were drawn at 0, 3, 6, and 12 months. Fasting glucose was measured using a hexokinase photometric assay, HbA1c was measured using turbidimetric inhibition immunoassay, and insulin was measured by electrochemiluminescence immunoassay (Roche Diagnostics). HOMA-IR was calculated according to the formula: [fasting insulin (microU/L) × fasting glucose (mmol/L)/22.5]. Lipid profile was measured at 0, 6, and 12 months. Total cholesterol, high-density lipoprotein cholesterol (HDL) and triglycerides were measured by enzymatic assay and low-density lipoprotein was calculated using the Friedewald equation (Roche Diagnostics). Other biochemistry assessments were performed using routine methodology for patient care at our tertiary academic center. Body weight and height were measured using standard anthropometric techniques. Waist to hip ratio was calculated at each study visit using the World Health Organization guidelines. Total and regional (android, gynoid, truncal) fat mass and lean mass were measured by DXA (Prodigy version 7.51; GE Lunar, Madison, Wisconsin, USA) at 0, 6, and 12 months. The coefficients of variation (CV), in our center, were less than 2% for repeated scans (31). Fat mass index was calculated according to the formula: [total fat mass (kg) / height (m)^2^].

### Statistical Analysis

Data are reported as mean (SD), median [Q1; Q3], or number (%), and crude differences were tested using Wilcoxon signed rank test or chi-square test in the case of frequencies. Statistical analysis of the treatment effect of denosumab relative to placebo over 12 months followed the intention-to-treat principle (ITT) and used repeated measures linear mixed effects models based on restricted maximum likelihood estimates (REML) (32). The models included fixed effects of baseline level, strata used for randomization (lumbar spine BMD and BMI), treatment group, study time points and the interaction of time points with the treatment group, and random effects at the subject level. Mixed models are robust against some observations missing-at-random, thereby allowing ITT analysis of all subjects that had been randomized. The treatment effect is reported as the mean adjusted difference (MAD) between the denosumab and placebo groups at 6 and 12 months, with a 95% profiled CI. The significance level was tested as a single *P* value between groups over all time points. A two-sided *P* value of < .05 was considered indicative of statistical significance. Results were not adjusted for multiple testing as outcomes were intercorrelated. As a sensitivity analysis, a per protocol analysis was additionally conducted, which included only subjects that adhered to the protocol and completed the trial. All statistical analyses were performed with the R statistical base package environment (version 4.3.1 for Mac) with the added packages lme4 1.1-34 and effects 4.2-2 (32-34).

### Role of the Funding Source

This was an investigator-sponsored study and there was no external input regarding the study design, data collection, data analysis, data interpretation, or writing of the report.

## Results

**Figure 1.**
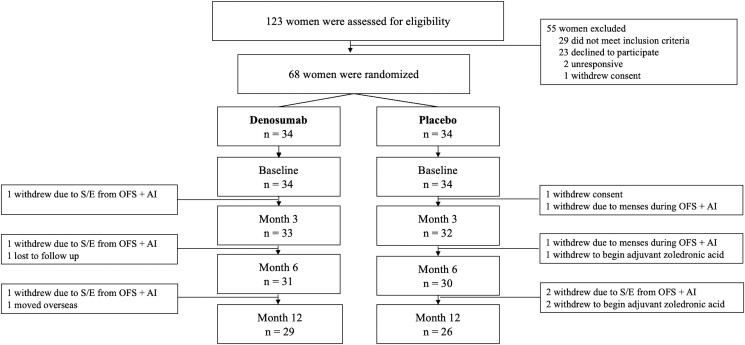
Trial profile. Abbreviations: AI, aromatase inhibition; OFS, ovarian function suppression; S/E, side effects.

Of the 117 women assessed for eligibility, 68 women were eligible and underwent randomization to denosumab 60-mg (n = 34) or placebo (n = 34). Fifty-nine participants (81%) completed the study (Fig. 1). All participants had evaluable DXA scans for measurement of fat and lean mass at each time point. Baseline characteristics are shown in Table 1.

**Table 1.** Baseline characteristics of the study participants

	Placebo (n = 34)	Denosumab (n = 34)	*P* value
Age, years	43.5 (37.8–48.4)	44.4 (41.8–47.2)	.75
Anthropometric measurements			
Weight, kg	67.8 (62.3–86.7)	70.3 (58.2–78.9)	.73
Body mass index, kg/m^2,^	24.9 (22.0–32.7)	24.9 (22.0–32.7)	.77
Waist circumference, cm	82.5 (77.8–97.8)	87.0 (75.8–95.8)	.99
Hip circumference, cm	104.0 (95.2–112.0)	102.0 (94.0–108.0)	.50
Waist:hip ratio	0.83 (0.78–0.90)	0.85 (0.77–0.91)	.65
Fat mass by DXA			
Total fat mass, g	25 499 (21 421–40 180)	25 972 (17 748–37 152)	.52
Android fat mass, g	2084 (1561–3087)	2080 (1486–3346)	.87
Gynoid fat mass, g	5482 (4634–7208)	5686 (4055–7122	.60
Truncal fat mass, g	12 212 (9985–20 272)	12 757 (8537–19 097)	.80
Fat mass index, kg/m^2^	9.28 (7.48–14.21)	9.81 (6.68–13.74)	.63
Lean mass by DXA			
Total lean mass, g	39 783 (35 895–45 867)	39 588 (36 065–42 856)	.80
Android lean mass, g	2785 (2424–3272)	2757 (2620–3038)	.77
Gynoid lean mass, g	5736 (5212–6532)	5646 (5303–6284)	.96
Truncal lean mass, g	19 258 (17 689–22 234)	19 236 (17 434–20 958)	.82
Glycemic parameters			
Glucose, mmol/L	4.90 (4.80–5.10)	4.90 (4.53–5.07)	.35
HbA1c, %	5.10 (4.90–5.50)	5.00 (4.82–5.20)	.36
Insulin, μU/L	8.90 (6.82–12.9)	7.50 (5.23–11.1)	.15
C-peptide, nmol/L	0.70 (0.55–0.98)	0.70 (0.54–0.89)	.85
HOMA-IR	1.99 (1.43–2.91)	1.61 (1.12–2.48)	.15
Lipid profile			
Total cholesterol, mmol/L	5.10 (4.50–5.80)	5.40 (5.03–5.77)	.10
LDL, mmol/L	3.10 (2.42–3.58)	3.25 (2.80–3.80)	.21
HDL, mmol/L	1.45 (1.21–1.65)	1.60 (1.31–1.90)	.12
Triglycerides, mmol/L	1.25 (0.90–1.67)	1.10 (0.80–1.50)	.32

Data are presented as median and interquartile range (Q1-Q3). Abbreviations: DXA, dual-energy x-ray absorptiometry; HDL, high-density lipoprotein cholesterol; HOMA-IR, homeostatic model assessment of insulin resistance; LDL, low-density lipoprotein cholesterol.

### Fat and Lean Mass by DXA

**Figure 2.**
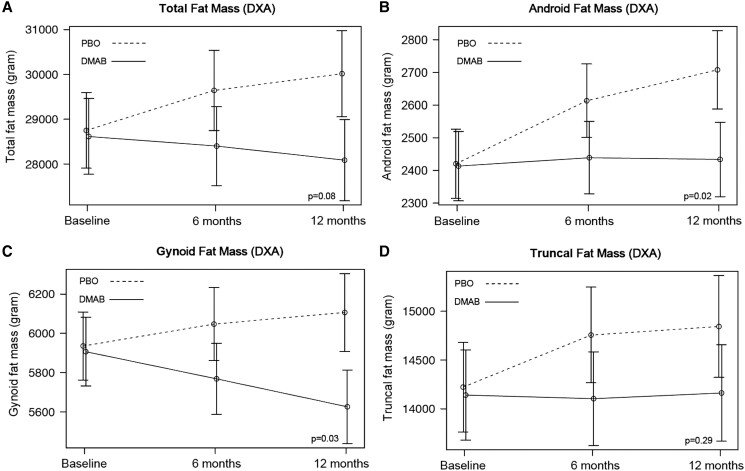
Mean adjusted differences (95% CI) in total (A) and regional (B-D) fat mass between the placebo group (PBO, dashed line) and denosumab group (DMAB, solid line) at 0, 6, and 12 months. The significance level was tested as a single *P* value over all time points. Abbreviation: DXA, dual-energy x-ray absorptiometry.

**Figure 3.**
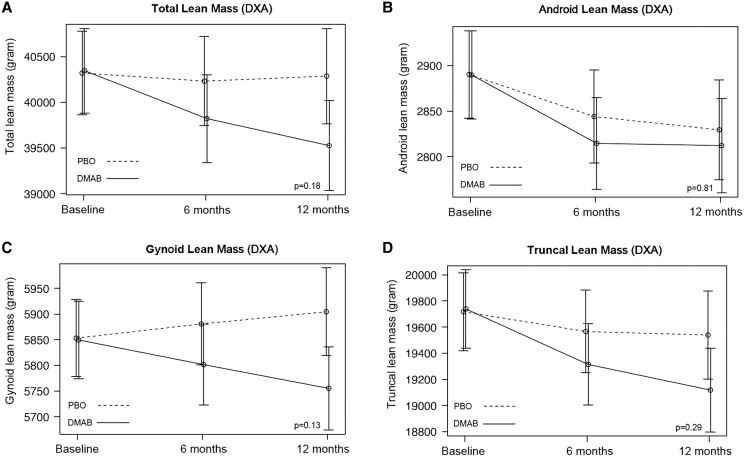
Mean adjusted differences (95% CI) in total (A) and regional (B-D) lean mass between the placebo group (PBO, dashed line) and denosumab group (DMAB, solid line) at 0, 6, and 12 months. The significance level was tested as a single *P* value over all time points. Abbreviation: DXA, dual-energy x-ray absorptiometry.

Over 12 months, relative to placebo, treatment with denosumab prevented the increase in both android (−266 [95% CI, −453 to −79], *P* = .02) and gynoid fat mass (−452 g [95% CI, −783 to −122], *P* = .03) (Table 2, Fig. 2). Similar effects were observed in total fat mass and fat mass index (−1792 g [95% CI, −3346 to −240], *P* = .08, and −0.64 kg/m^2^ [95% CI, −1.22 to −.06], *P* = .09, respectively); neither achieved statistical significance at the .05 cutoff. There was no major effect on truncal fat mass (−600 g [95% CI, −1452 to −251], *P* = .29). Over 12 months, no significant treatment effect was observed on measures of total or regional lean mass (Table 2, Fig. 3).

**Table 2.** Anthropometric measurements and total and regional fat and lean mass (intention-to-treat)

	Placebo (n = 34)	Denosumab (n = 34)	Mean adjusted difference (95% CI)	Overall *P* value
Weight, kg				.23
0 months	67.8 (62.3–86.7)	70.3 (58.2–78.9)		
3 months	69.6 (63.1–86.2)	70.2 (56.8–80.0)	−0.57 [−2.27 to 1.12]	
6 months	68.8 (61.4–84.9)	70.2 (57.4–80.1)	−1.10 [−2.83 to .63]	
12 months	69.2 (63.2–87.8)	67.3 (56.7–81.9)	−1.83 [−3.60 to 0.07]	
Body mass index, kg/m^2^				.19
0 months	24.9 (22.0–32.7)	24.9 (22.4–30.1)		
3 months	24.8 (23.2–31.9)	25.1 (22.3–30.7)	−0.25 [−0.89 to 0.39]	
6 months	24.4 (22.7–30.9)	26.1 (22.5–31.1)	−0.52 [−1.18 to 0.14]	
12 months	24.9 (23.3–31.6)	24.0 (22.3–31.7)	−0.71 [−1.38 to −0.04]	
Waist circumference, cm				.06
0 months	82.5 (77.8–97.8)	87.0 (75.8–95.8)		
3 months	85.0 (79.0–96.5)	88.0 (80.0–94.0)	−0.09 [−2.96 to 2.78]	
6 months	87.0 (79.0–92.0)	88.0 (75.5–91.5)	−1.62 [−4.55 to 1.30]	
12 months	87.0 (78.2–96.8)	83.5 (73.0–93.5)	−3.77 [−6.76 to −0.79]	
Hip circumference, cm				.73
0 months	104.0 (95.2–112.0)	102.0 (94.0–108.0)		
3 months	102.0 (98.5–114.0)	102.0 (95.0–109.0)	−0.57 [−3.18 to 2.04]	
6 months	102.0 (98.0–110.0)	101.0 (92.5–108.0)	−0.99 [−3.66 to 1.67]	
12 months	102.0 (97.2–113.0)	98.5 (90.2–108.0)	−1.55 [−4.29 to 1.17]	
Waist to hip ratio				.29
0 months	0.83 (0.78–0.90)	0.85 (0.77–0.91)		
3 months	0.84 (0.78–0.88)	0.84 (0.80–0.88)	0.00 [−0.02 to 0.03]	
6 months	0.85 (0.79–0.88)	0.86 (0.81–0.89)	−0.00 [−0.03 to 0.03]	
12 months	0.85 (0.82–0.90)	0.85 (0.80–0.89)	−0.02 [−0.05 to 0.01]	
Total fat mass, g				.08
0 months	25 499 (21 421–40 180)	25 972 (17 748–37 152)		
6 months	26 600 (20 352–37 006)	26 546 (17 782–35 994)	−1105 [−2606 to 398]	
12 months	28 004 (21 190–37 701)	25 850 (17 973–39 267)	−1792 [−3346 to −24]	
Android fat mass, g				.02
0 months	2084 (1561–3087)	2080 (1486–3346)		
6 months	1198 (1434–2979)	2342 (1310–3372)	−167 [−347 to 14]	
12 months	2306 (1578–3462)	2312 (1618–3361)	−266 [−453 to −79]	
Gynoid fat mass, g				.03
0 months	5482 (4634–7208)	5686 (4055–7122)		
6 months	5717 (4420–7062)	5815 (3820–7054)	−250 [−570 to 71]	
12 months	5658 (4624–7246)	5510 (3931–6969)	−452 [−783 to −122]	
Truncal fat mass, g				.29
0 months	12 212 (9985–20 272)	12 757 (8537–19 097)		
6 months	11 867 (9363–17 661)	12 948 (8020–18 818)	−571 [−1395 to 253]	
12 months	13 126 (9753–19 851)	13 091 (9331–19 087)	−600 [−1452 to 251]	
Fat mass index, kg/m^2^				.09
0 months	9.28 (7.48–14.21)	9.81 (6.68–13.74)		
6 months	9.01 (8.07–14.02)	10.17 (6.48–13.75)	−0.44 [−1.00 to 0.12]	
12 months	9.83 (8.18–13.98)	10.35 (6.93–14.49)	−0.64 [−1.22 to −0.06]	
Total lean mass, g				.18
0 months	39 783 (35 895–45 867)	39 588 (36 065–42 856)		
6 months	39 760 (35 439–43 882)	39 651 (35 794–41 128)	−435 [−1231 to 361]	
12 months	39 218 (36 274–45 664)	39 167 (35 521–40 787)	−785 [−1610 to 37]	
Android lean mass, g				.81
0 months	2785 (2424–3272)	2757 (2620–3038)		
6 months	2751 (2477–3166)	2710 (2438–2957)	−29 [−116 to 59]	
12 months	2782 (2442–3032)	2701 (2483–2869)	−17 [−107 to 74]	
Gynoid lean mass, g				.13
0 months	5736 (5212–6532)	5646 (5303–6284)		
6 months	5768 (5198–6470)	5601 (5337–6200)	−76 [−209 to 58]	
12 months	5753 (5229–6512)	5582 (5261–6027)	−146 [−284 to −9]	
Truncal lean mass, g				.29
0 months	19 258 (17 689–22 234)	19 236 (17 434–20 958)		
6 months	18 930 (17 688–21 457)	18 834 (16 958–20 590)	−273 [−805 to 258]	
12 months	19 250 (17 415–21 847)	18 425 (17 250–19 599)	−441 [−994 to 105]	

Data are presented as median (interquartile range, Q1-Q3). The treatment effect is reported as the mean adjusted difference and its 95% CI between the placebo and denosumab groups using repeated measures linear mixed effects models. The significance level was tested as a single *P* value over all time points (overall *P* value).

### Anthropometric Measures, Glucose Metabolism, and Lipid Profile

**Figure 4.**
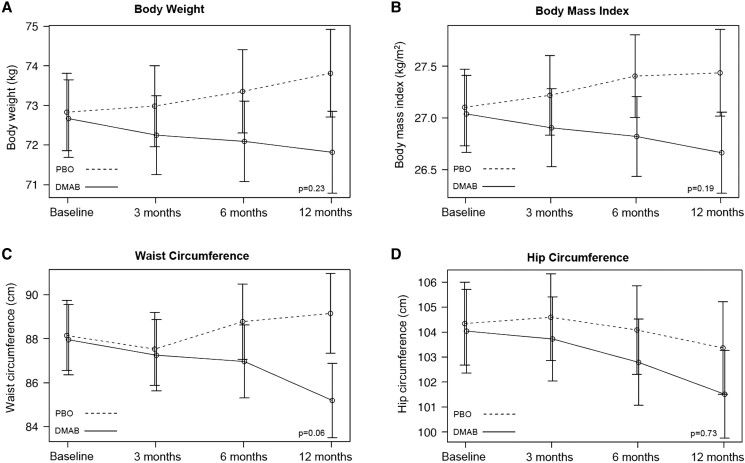
Mean adjusted differences (95% CI) in anthropometric measurements between the placebo group (PBO, dashed line) and denosumab group (DMAB, solid line) at 0, 6, and 12 months. The significance level was tested as a single *P* value over all time points.

Relative to the placebo group, waist circumference was lower in the denosumab group but did not achieve statistical significance (−3.77 cm [95% CI, −6.76 to −.79], *P* = .06). There was no significant treatment effect observed on body weight, BMI, hip circumference, waist to hip ratio, fasting blood glucose, HbA1c, fasting insulin, C-peptide concentrations, HOMA-IR, or fasting lipid profile (Tables 2 and 3, Fig. 4).

**Table 3.** Glucose metabolism and lipid parameters (intention-to-treat)

	Placebo (n = 34)	Denosumab (n = 34)	Mean adjusted difference (95% CI)	Overall *P* value
Glucose, mmol/L				.31
0 months	4.90 (4.80–5.10)	4.90 (4.53–5.07)		
3 months	4.80 (4.60–5.15)	4.90 (4.50–5.10)	0.15 [−0.09 to 0.40]	
6 months	4.85 (4.50–5.10)	4.80 (4.50–5.15)	0.07 [−0.18 to 0.32]	
12 months	5.00 (4.70–5.25)	4.85 (4.60–5.18)	−0.09 [−0.35 to 0.17]	
HbA1c, %				.53
0 months	5.10 (4.90–5.50)	5.00 (4.82–5.20)		
3 months	5.40 (5.20–5.57)	5.30 (5.00–5.60)	0.02 [−0.16 to 0.20]	
6 months	5.30 (5.10–5.50)	5.20 (5.05–5.45)	0.07 [−0.11 to 0.25]	
12 months	5.30 (5.20–5.50)	5.35 (5.20–5.60)	0.13 [−0.05 to 0.31]	
Insulin, mU/L				.83
0 months	8.90 (6.82–12.9)	7.50 (5.23–11.1)		
3 months	9.35 (5.73–12.0)	9.50 (5.70–13.0)	1.60 [−1.87 to 5.07]	
6 months	8.30 (6.10–14.6)	8.30 (5.20–13.9)	1.03 [−2.46 to 4.54]	
12 months	7.80 (5.45–13.0)	7.95 (5.70–10.5)	0.49 [−3.09 to 4.06]	
C-peptide, nmol/L				.94
0 months	0.73 (0.55–0.98)	0.70 (0.54–0.89)		
3 months	0.76 (0.63–0.92)	0.76 (0.56–0.90)	−0.04 [−0.18 to 0.10]	
6 months	0.72 (0.56–0.95)	0.62 (0.51–0.99)	−0.04 [−0.18 to 0.11]	
12 months	0.62 (0.56–0.86)	0.68 (0.54–0.82)	−0.29 [−0.17 to 0.12]	
HOMA-IR				.57
0 months	1.99 (1.43–2.91)	1.61 (1.12–2.48)		
3 months	2.12 (1.16–2.73)	2.07 (1.18–2.54)	0.57 [−0.29 to 1.44]	
6 months	1.74 (1.37–3.13)	1.70 (1.06–3.03)	0.39 [−0.48 to 11.27]	
12 months	1.66 (1.24–3.01)	1.75 (1.22–2.42)	0.10 [−0.79 to 1.00]	
Total cholesterol, mmol/L				.40
0 months	5.10 (4.50–5.80)	5.40 (5.03–5.77)		
6 months	5.15 (4.43–5.77)	5.70 (4.85–6.10)	0.15 [−0.18 to 0.47]	
12 months	5.30 (4.45–5.85)	5.40 (4.73–6.07)	−0.09 [−0.42 to 0.24]	
LDL, mmol/L				.48
0 months	3.10 (2.42–3.58)	3.25 (2.80–3.80)		
6 months	2.90 (2.50–3.60)	3.40 (2.90–4.00)	0.13 [−0.15 to 0.42]	
12 months	3.40 (2.75–3.70)	3.40 (2.82–3.88)	−0.05 [−0.34 to 0.25]	
HDL, mmol/L				.89
0 months	1.45 (1.21–1.65)	1.60 (1.31–1.90)		
6 months	1.50 (1.30–1.64)	1.50 (1.30–1.80)	−0.03 [−0.14 to 0.09]	
12 months	1.40 (1.21–1.67)	1.50 (1.33–1.87)	−0.02 [−0.14 to 0.09]	
Triglycerides, mmol/L				.25
0 months	1.25 (0.90–1.67)	1.10 (0.80–1.50)		
6 months	1.10 (0.70–1.37)	1.00 (0.70–1.45)	0.17 [−0.04 to 0.39]	
12 months	1.20 (0.85–1.50)	0.85 (0.70–1.30)	0.02 [−0.20 to 0.25]	

Data are presented as median (interquartile range, Q1-Q3). The treatment effect is reported as mean adjusted difference and its 95% CI between the placebo and denosumab groups using repeated measures linear mixed effects models. The significance level was tested as a single *P* value over all time points (overall *P* value). Abbreviations: HOMA-IR, homeostatic model assessment of insulin resistance; LDL, low-density lipoprotein cholesterol; HDL, high-density lipoprotein cholesterol.

In a sensitivity per protocol analysis conducted only on participants who completed the study and adhered to the study protocol (n = 55), outcomes were very similar to the main ITT analysis (data not shown).

## Discussion

In this prespecified secondary analysis of a double-blind RCT, we report that treatment with denosumab at the time of initiating maximal estradiol suppression in premenopausal women with ER-positive breast cancer prevented the increase in some measures of fat mass, compared with the placebo group. This metabolically favorable change did not translate into improvements in glucose or lipid measurements, nor were there detectable changes in lean mass.

The menopause transition is associated with an increase in the distribution of adipose tissue predominantly around the abdomen (subcutaneous and visceral fat) and trunk (android fat mass distribution pattern) (35, 36). It appears that the regional distribution of body fat, rather than excess body fat *per se*, is a more important determinant of cardiovascular risk (37). Indeed, fat accumulation in an android distribution is significantly associated with a higher cardiovascular risk and progression to type 2 diabetes (38-42). In this study, denosumab prevented the increase in android fat mass, compared with the placebo group.

While there was a detectable between-group treatment effect on android and gynoid fat mass over 12 months, we did not achieve a statistically significant between-group treatment effect on total fat mass, BMI, body weight, or waist circumference. However, the respective 95% CIs, [−3346 to −24 g], [−1.38 to −0.04 kg/m^2^], [−3.60 to 0.07 kg], and [−6.76 to −0.79 cm], were consistent with a trend toward a decrease in these parameters in women treated with denosumab compared to placebo.

To our knowledge, there have been no published prospective placebo-controlled RCTs evaluating the effect of denosumab on fat mass. In a cohort of postmenopausal women with diabetes who were receiving denosumab (n = 115), bisphosphonates (n = 115), or calcium and vitamin D (n = 115), treatment with denosumab was associated with a ∼1.2% (*P* < .0001) and ∼0.8% (*P* = .008) decrease in BMI at 12 months compared with the bisphosphonate and calcium and vitamin D groups, respectively (15). However, the clinical significance of these relatively small differences in BMI is uncertain. Although RANKL and osteoprotegerin (OPG), a decoy receptor with high affinity for RANKL and a natural analogue of denosumab, are expressed in adipose tissue (43, 44), mechanistic evidence examining the effects of denosumab on adipose tissue is lacking.

We were unable to detect improvements in glucose metabolism or lipid profiles. These findings are consistent with previous observational studies in postmenopausal women without diabetes (26-28). In addition, in a post hoc analysis of the FREEDOM study, a 3-year randomized placebo-controlled study evaluating the efficacy of denosumab on fracture risk reduction, treatment with denosumab did not improve glucose homeostasis in postmenopausal women with prediabetes or those with diabetes receiving antidiabetic treatment (25). By contrast, several studies suggest that denosumab therapy may be associated with improved glucose homeostasis, but primarily in women with evidence of prediabetes or diabetes. In the same post hoc analysis of the FREEDOM study, denosumab was associated with a modest reduction in fasting plasma glucose in postmenopausal women with diabetes who were not receiving antidiabetic medication (25). In addition, in a 2.2-year observational cohort study of 4301 new users of denosumab and 21 038 users of an oral bisphosphonate, obtained from a primary care database, treatment with denosumab was associated with a reduced incidence of type 2 diabetes, which was more evident in those with prediabetes and a BMI of >30 kg/m^2^ (22). In general, most studies point toward a favorable effect of denosumab on glucose homeostasis in women with some degree of impaired glucose tolerance, with minimal or no effects observed in women with normal glucose tolerance.

This study has several limitations. While secondary outcomes were prespecified, the study was only 12 months in duration and may have been underpowered to detect smaller treatment effects in some glucose metabolism or lipid parameters. Moreover, we excluded women with established diabetes in whom such changes might have become apparent more readily. We did not collect data on food intake or objectively monitor physical activity. While the menopause transition is associated with unfavorable metabolic changes, the changes in metabolic parameters observed in this study cannot be attributed solely to treatment with ovarian function suppression and aromatase inhibition, as there may be other contributing factors that are unaccounted for. The effects of denosumab reported here may not be generalizable to other populations, as participants in our study were premenopausal women without diabetes commencing estradiol suppression therapy for breast cancer.

In summary, in premenopausal women with ER-positive early-stage breast cancer commencing maximal estradiol suppression, treatment with denosumab prevented the increase in gynoid and android fat mass, with trends observed in preventing the increase in total fat mass and waist circumference. The leading cause of death in breast cancer survivors is cardiovascular disease, not the cancer itself (10), and adverse metabolic changes may contribute to this risk. As such, further longer-term prospective clinical studies are needed to determine the clinical relevance of these findings. Whether these adverse metabolic changes associated with estradiol suppression may be mitigated by denosumab therapy also requires further study.

## Data Availability

All datasets generated and/or analyzed during the current study are not publicly available but are available from the corresponding author on reasonable request.

## Clinical Trial Information

The study was preregistered with the Australian New Zealand Clinical Trials Registry (anzctr.org.au), identifier ACTRN12616001051437.

## Abbreviations

BMD: bone mineral density
BMI: body mass index
DXA: dual-energy x-ray absorptiometry
ER: estrogen receptor
HbA1c: glycated hemoglobin
HOMA-IR: homeostatic model assessment for insulin resistance
ITT: intention to treat
RANKL: receptor activator of nuclear factor kappa-B ligand
RCT: randomized controlled trial
